# Angioinvasive Rhino-Orbital-Cerebral Mucormycosis in a Patient with Type 2 Diabetes Mellitus: A Complex and Lethal Cause of Stroke

**DOI:** 10.3390/diagnostics14192246

**Published:** 2024-10-08

**Authors:** Nadine Weissert, Annerose Mengel, Katharina Bader, Florian Hennersdorf, Katharina Feil

**Affiliations:** 1 Department of Neurodegenerative Disease, University Hospital Tübingen, Eberhard Karls University, 72076 Tübingen, Germany; nadine.weissert@med.uni-tuebingen.de; 2 Department of Neurology and Stroke, University Hospital Tübingen, Eberhard Karls University Tübingen, 72076 Tübingen, Germany; katharina.feil@uni-tuebingen.de; 3 Department of Otolaryngology, Head and Neck Surgery, University Hospital Tübingen, Eberhard Karls University Tübingen, 72076 Tübingen, Germany; katharina.bader@med.uni-tuebingen.de; 4 Department of Diagnostic and Interventional Neuroradiology, University Hospital Tübingen, Eberhard Karls University, 72076 Tübingen, Germany; florian.hennersdorf@med.uni-tuebingen.de

**Keywords:** rhino-orbital-cerebral mucormycosis, type 2 diabetes mellitus, stroke, MRI, angiography, intracranial internal carotid artery stenosis, ocular protrusion, periorbital enhancement

## Abstract

Rhino-orbital-cerebral mucormycosis is a rapidly progressive and often fatal fungal infection caused by molds of the order *Mucorales*, particularly affecting immunocompromised individuals. This infection is notorious for its angioinvasive properties, enabling the fungi to invade blood vessels and leading to tissue necrosis. We report the clinical course of a 59-year-old Caucasian man with poorly controlled type 2 diabetes (HbA1c 16.8%) who presented with unilateral headache, left-sided facial numbness, and incomplete left ocular motor paresis. Initial presentation raised suspicion of orbital phlegmon, leading to antibiotic and later corticosteroid pulse therapy, which worsened the patient’s condition. Subsequent imaging demonstrated extensive inflammatory changes, including wall irregularities of the left intracranial internal carotid artery, accompanied by ocular protrusion and periorbital enhancement. New palatal lesions indicated mucormycosis, which was confirmed by molecular analysis of a palatal biopsy, leading to Amphotericin B treatment. Pre-surgery imaging revealed a malignant middle cerebral artery infarction, and the patient died 16 days after symptom onset and 12 days after initial presentation under palliative care due to a poor prognosis. This case of angioinvasive mucormycosis underscores the severe and often fatal course of rhino-orbital-cerebral mucormycosis in an immunocompromised individual. The rapid progression from initially vague and unspecific symptoms to extensive vascular involvement and stroke highlights the critical need for early and accurate diagnosis, as well as prompt intervention to prevent further disease progression. Additionally, this case also illustrates the potential risks associated with corticosteroid therapy in the presence of undiagnosed fungal infections, which can exacerbate the condition and lead to serious complications. Clinicians should maintain a high index of suspicion for mucormycosis in similar clinical scenarios, prioritizing adequate antifungal treatment and careful monitoring to improve patient outcomes. Early interdisciplinary collaboration is essential for the effective management of such complex cases.

We report the clinical course of a 59-year-old Caucasian man with poorly controlled type 2 diabetes (HbA1c 16.8%). The patient initially presented with a unilateral headache and left-sided facial numbness as well as diffuse soft tissue swelling of the face, particularly around the left orbit (Figure 1A–C). The symptoms began approximately 3–4 days before the initial presentation at the emergency department of an external clinic, where the patient was seen due to an acute and severe exacerbation of the headache. An initial computed tomography (CT) with angiography (CTA) was performed, which was unremarkable regarding brain parenchyma, aside from newly noted calcification in the basal ganglia compared to prior imaging two years before (Figure 2A). Subsequent CTA, performed with a mixed arterial and venous phase (Figure 2B), demonstrated intracranial and supra-aortic arteries without significant stenosis or occlusion. A finding was a stenosis at the origin of the left vertebral artery, attributed to a calcified plaque. The scan also revealed several contrast-enhancing lymph nodes, up to 9 mm in size, bilaterally in the neck, which were described as unspecific. Importantly, there was no evidence of sinus or cerebral venous thrombosis.

A complementary lumbar puncture was performed due to the persistent headaches. The cerebrospinal fluid (CSF) revealed no pleozytosis (2/3 lymphocytic cells), a slightly elevated protein level of 61.7 mg/dL (reference range 5–40 mg/dL) as well as elevated lactate levels at 3.8 mmol/L (reference range: 0.9–2.7 mmol/L), and glucose of 247 mg/dL with a corresponding serum glucose level of 433 mg/dL. Interleukin-6 (IL-6) was significantly elevated in the CSF at 208 pg/mL (reference range: 0–7 pg/mL). Further infectious diagnostics from the CSF were not pursued.

Serology for syphilis, bartonellosis, and borreliosis was negative, while toxoplasmosis serology indicated a subacute infection with high IgM avidity. Herpes simplex virus 1/2 (HSV-1/2) and varicella-zoster virus (VZV) IgM and IgG were negative, while cytomegalovirus (CMV) IgG was positive. Tests for Human Immunodeficiency Virus (HIV), Hepatitis B surface antigen (HBsAg), antibody to Hepatitis B surface antigen (anti-HBs), antibody to Hepatitis B core antigen (anti-HBc), and Hepatitis C virus (HCV) were all negative.

Due to clinical signs and elevated CRP levels (21 mg/dL), an initial diagnosis of orbital phlegmon was considered. Orbital phlegmon, a bacterial infection that affects the tissues surrounding the eye, can result from sinusitis or direct trauma and may lead to severe complications if not treated promptly [1,2].

In our patient, antibiotic therapy with Piperacillin/Tazobactam was initiated, and switched to Ceftriaxzone intravenously three days later. Due to the initial suspicion of cavernous sinus thrombosis, heparin therapy was started but later discontinued after imaging excluded thrombosis (Figure 2B) [3]. Progressive and severe headaches persisted, with only moderate relief from analgesics such as tilidin, metamizole, and piritramid.

In the dermatological assessment, the soft tissue swelling, now with skin changes, was described to be most consistent with eczema (Figure 1A–C). Both upper eyelids (more pronounced on the left side) displayed confluent, poorly demarcated erythematous maculae with adherent pityriasiform scaling and mild lichenification. Similar changes were noted on the neck, with generalized sebostasis, without petechiae, erythematous plaques, pustules, or ulcerations.

**Figure 2:**
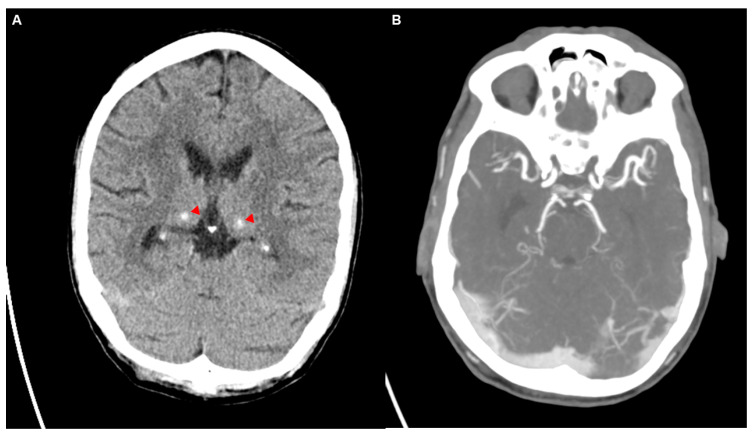
The initial non-contrast CT scan of the head (**A**) revealed cerebrospinal fluid spaces that were age-appropriate, with no evidence of mass effect or intracranial hemorrhage. There were no signs of acute ischemic changes. Hyperdensities observed bilaterally in the thalami were consistent with calcifications, which were newly noted compared to prior imaging from 2022 (two years before). Additionally, defects that were previously observed in the right temporal lobe on imaging two years earlier were noted again, accompanied by ex vacuo dilation of the right temporal horn of the lateral ventricle. The calcifications of the basal ganglia, although newly observed, were considered incidental and had no clinical significance, consistent with previous reports of age-related or physiological calcifications in the basal ganglia and thalamus [4]. A CT angiography (CTA) (**B**) of the extra- and intracranial vessels in a mixed arterial and venous phase. There was a calcified plaque in the left vertebral artery, besides that the CTA showed normal arterial and venous structures without any evidence of vascular malformations, stenoses, or aneurysms. There are no signs of thrombosis or other vascular abnormalities, with intact and well-defined vessels throughout the visible cerebral vasculature.

Two days after initial presentation, the patient exhibited incomplete ocular motor paresis, which progressively worsened over the next three days, finally leading to a complete left-sided opthalmoplegia with involvement of the oculomotorius (III), trochlear (IV), and abducens (VI) cranial nerves. Clinically, the patient had decreased visual acuity, and a fixed and dilated pupil on the left side. In the ophthalmological evaluation, the right eye was largely normal, with a visual acuity of 0.3, a clear cornea, regular eyelids, and no signs of retinal pathology. There was no relative afferent pupillary defect (RAPD), and ocular motility was unrestricted. Conversely, the left eye exhibited significant pathology, with pronounced swelling and redness extending across the nose and supraorbital region (as described in Figure 1). The conjunctiva was chemotic, the pupil was minimally reactive, and there was optic disc swelling with central retinal edema. CT imaging of the orbit and sinuses were performed (Figure 3). There was no imaging or clinical-endoscopic evidence to support an orbital complication arising from sinusitis, particularly ethmoiditis. The inflammatory-edematous swelling appeared to extend predominantly to the superficial layers of the eyelids and the frontal and temporal facial skin, rather than indicating a deeper orbital or sinus origin. Considering the patient’s history of antibody-negative autoimmune encephalitis two years before, there was suspicion of an IgG-associated autoimmune process. A follow-up consultation with the ophthalmology department led to the recommendation of corticosteroid therapy with 250 mg of prednisolone twice daily in addition to antibiotic treatment, and a clinical re-evaluation [5].

Under corticosteroid therapy, there was no improvement in the patient’s symptoms, particularly the local swelling, and the complete ophthalmoplegia persisted. Instead, following the initiation of corticosteroids, the patient developed additional neurological symptoms, including fluctuating episodes of aphasia as well as mild right-sided hemiparesis (National Institutes of Health Stroke Scale [NIHSS] 7 points) starting on day 3 of the treatment. Therefore, cerebral CT with CTA was repeated and showed no significant changes compared to the initial scan on the day of admission (as described in Figure 2A,B), except that the soft tissue swelling appeared to be more pronounced now. Additional cerebral magnetic resonance imaging (MRI) did not reveal any diffusion restrictions. Multiple small white matter lesions were identified bilaterally in the periventricular regions, likely indicative of chronic microangiopathic changes. Symmetrical calcifications in the thalami were consistent with findings from the previous CT scan. The MRI also confirmed the right temporal defect with ex vacuo dilation of the temporal horn. Regarding the local symptoms, venous dilation in the left superior ophthalmic vein was noted, along with significant phlegmonous enhancement at the apex of the left orbit, involving the left superior rectus muscle and potentially the optic nerve. Adjacent sinusitis was observed in the left frontal, ethmoid, sphenoid, and maxillary sinuses, accompanied by mild swelling of the left eyelid and inflammation of the adjacent subcutaneous tissue as well as contrast-enhanced protrusion of the left eye muscles (Figure 4).

The patients experienced further clinical deterioration marked by decreased vigilance, worsening aphasia, and intensified right-sided hemiparesis, resulting in an NIHSS of 15 points. On day 7 after initial presentation at the first hospital (day 11 after symptom onset), the patient was transferred to our university clinic and admitted to our neurological intensive care unit (NICU). MRI was repeated, showing unchanged local inflammatory changes; however, new vessel abnormalities as well as diffusion restriction in the left middle cerebral artery territory were observed (Figure 5A,B). Based on these findings and the differential diagnosis of vasculitis or vasospasm, a digital subtraction angiography (DSA) was performed (Figure 5C).

Upon admission to our NICU, corticosteroid therapy was immediately discontinued. A repeated lumbar puncture revealed pleocytosis with 31 cells (67% mononuclear cells), a slightly elevated lactate level of 3.0 mmol/L (reference range: 0–2.2 mmol/L), and a protein concentration of 58 mg/dL (reference range: 0–45 mg/dL), indicating blood–brain barrier disruption. The glucose CSF/serum quotient was 0,7, with glucose levels of 185 mg/dL in the CSF and 433 mg/dL in the serum. No intrathecal IgG production was observed, and the IgG index was within the normal range at 0.45. Further CSF analysis included ferritin (0.6 µg/dL) and albumin (311 mg/L). IL-6 levels again were significantly elevated at 247.9 ng/L (reference range: 0–24 ng/L), consistent with a robust inflammatory response.

Extensive pathogen diagnostics of CSF were negative. No bacteria, fungi, or viruses were detected. Specific PCR tests for *Neisseria meningitidis*, *Streptococcus pneumoniae*, *Listeria monocytogenes*, *Streptococcus agalactiae*, *Haemophilus influenzae*, and *Escherichia coli* were negative, as were fungal PCR and cultures. Molecular testing also showed no detectable viral DNA, including HSV-1/2, VZV, CMV, EBV, and HHV-6. Additionally, no *Toxoplasma gondii* DNA was detected. The fungal PCR included common pathogens such as *Aspergillus* spp., *Candida* spp., *Pneumocystis jirovecii*, and *Cryptococcus* spp. The CSF cultures were negative for both bacteria and fungi after 48 h, and no further growth was observed after five days of incubation.

Further autoimmune and paraneoplastic antibody testing, including Anti-HU, Anti-RI, Anti-YO, Anti-MA, and Anti-NMDA receptor antibodies were negative in both serum and CSF.

The patient’s inflammatory markers were elevated, with CRP at 14.8 mg/dL, Procalcitonin at 0.11 ng/mL, and serum IL-6 at 77.6 ng/L. The patient was cardiopulmonary stable. Serum analysis showed no evidence of active HSV-1/2 or CMV infection, although VZV IgG was positive, suggesting previous exposure. Repeated blood cultures were negative for bacteria and fungi after five days of incubation. The Quantiferon test for *Mycobacterium tuberculosis* was inconclusive, likely due to impaired cellular immunity or improper handling. Further tests for direct pathogen detection were recommended. Candida antigen testing was borderline positive at 86.7 pg/mL, raising the possibility of invasive candidiasis, with follow-up testing recommended in 1–2 days. Aspergillus antigen testing (galactomannan) was negative, ruling out invasive aspergillosis.

Antibiotic therapy was escalated to Meropenem, Vancomycin, and Metronidazole. Initially, there was a slight improvement in swelling and inflammatory markers under this antibiotic therapy, but the patient’s clinical condition continued to deteriorate.

A repeated ENT evaluation conducted after NICU admission (day 8 after initial presentation in the external hospital and day 12 after symptom onset) concluded that an inflammatory focus in the paranasal sinuses as the cause of the patient’s symptoms was still discussed to be highly unlikely. There was no radiological or clinical-endoscopic evidence of an orbital complication of ethmoid sinusitis. During this ENT examination (as well as clinical examinations after NICU admission), the palate appeared unremarkable. A nasal mucosal biopsy from the nasal septum had been planned for the following day.

However, on day 9 after initial presentation and day 13 of symptom onset, new necrotic lesions appeared on the palate, indicative of severe tissue invasion and necrosis, consistent with invasive fungal infection, likely mucormycosis (Figure 6). These developments necessitated the initiation of Amphotericin B treatment [6].

An immediate biopsy of the palatal lesion was performed that day, revealing histological findings of multiple fragments overlaid with squamous epithelium transitioning to ulcerated areas with necrosis and florid inflammation. Numerous fungal hyphae of varying sizes were observed within these fragments, with most appearing non-septate and relatively thick. Several hyphae showed angioinvasion. Additionally, other septate hyphae with fewer spores were also noted. PAS staining highlighted these fungal hyphae, though they were more distinct in the HE staining and did not resemble Candida, despite being strongly PAS-positive. The Grocott stain showed minimal fungal hyphae in the deeper sections.

Following molecular analysis, the sample tested positive for *Mucor* spp. DNA sequences. Direct DNA sequencing confirmed the presence of *Rhizopus* spp., although a more specific classification was not possible. PCR tests for *Aspergillus* spp., *Candida* spp., *Pneumocystis jirovecii*, and *Cryptococcus* spp. were negative. Both the extraction control and external positive controls were correctly represented.

These findings confirmed the suspected diagnosis of mucormycosis, specifically caused by *Rhizopus* spp., and underscored the need for continued intensive monitoring and adequate antifungal therapy in the ICU setting.

Clinically, the patient’s condition remained poor despite all interventions. A surgical intervention was planned in collaboration with the ENT and ophthalmology teams. However, during preoperative imaging (Figure 7), a malignant MCA infarction with significant midline shift of approximately 1 cm was revealed corresponding to a newly demarcated infarction across the entire left MCA territory. Given the severity of these findings and in alignment with the presumed wishes of the patient, a decision was made to shift the focus of care to palliative treatment. The patient died 12 days after initial presentation and 16 days after symptom onset.

This case highlights the diagnostic and therapeutic challenges associated with atypical infections in immunocompromised patients, especially those with poorly controlled diabetes. The rapid progression of the disease, and the difficulties in achieving an early diagnosis, underscore the importance of heightened vigilance and cautious treatment choices in similar cases.

## Figures and Tables

**Figure 1:**
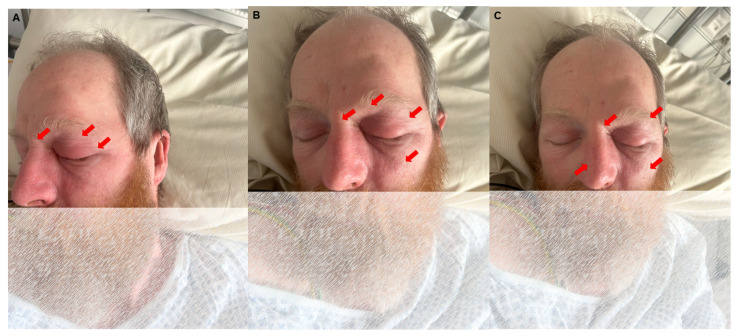
The patient initially presented with a unilateral headache and left-sided facial numbness as well as diffuse soft tissue swelling of the face, particularly around the left orbit (**A**–**C, red arrows**). The swelling is characterized by a reddish hue, extending diffusely around the orbit with no distinct or well-demarcated borders.

**Figure 3:**
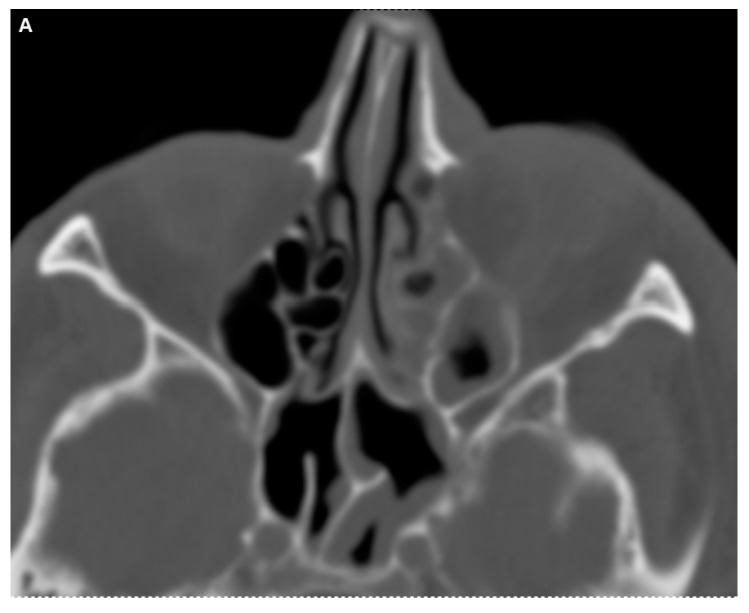

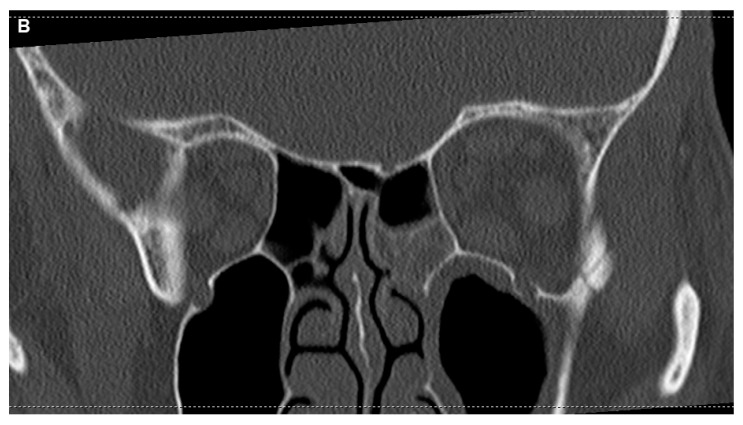
CT imaging of the orbit and paranasal sinuses. The axial view reveals soft tissue swelling of the left periorbital region, with both globes appearing intact. There was no evidence of intra- or extraconal hematoma or abscess. Notably, increased vascular markings and fat imbibition within the left orbit as well as swelling of the external orbital muscles were visible, suggesting inflammatory changes. Mucosal swelling was present in the paranasal sinuses; however, the bone structures were not affected at all (**A**). A coronal view shows partial opacificagion of the left sphenoid sinus, which retinas some aeration. Despide this, following further consultation with ENT specialists, it was considered unlikely that the paranasal sinuses were the primary source of the patient’s symptoms. While partial opacification of the left sphenoid sinus was observed, it remained partially aerated.

**Figure 4:**
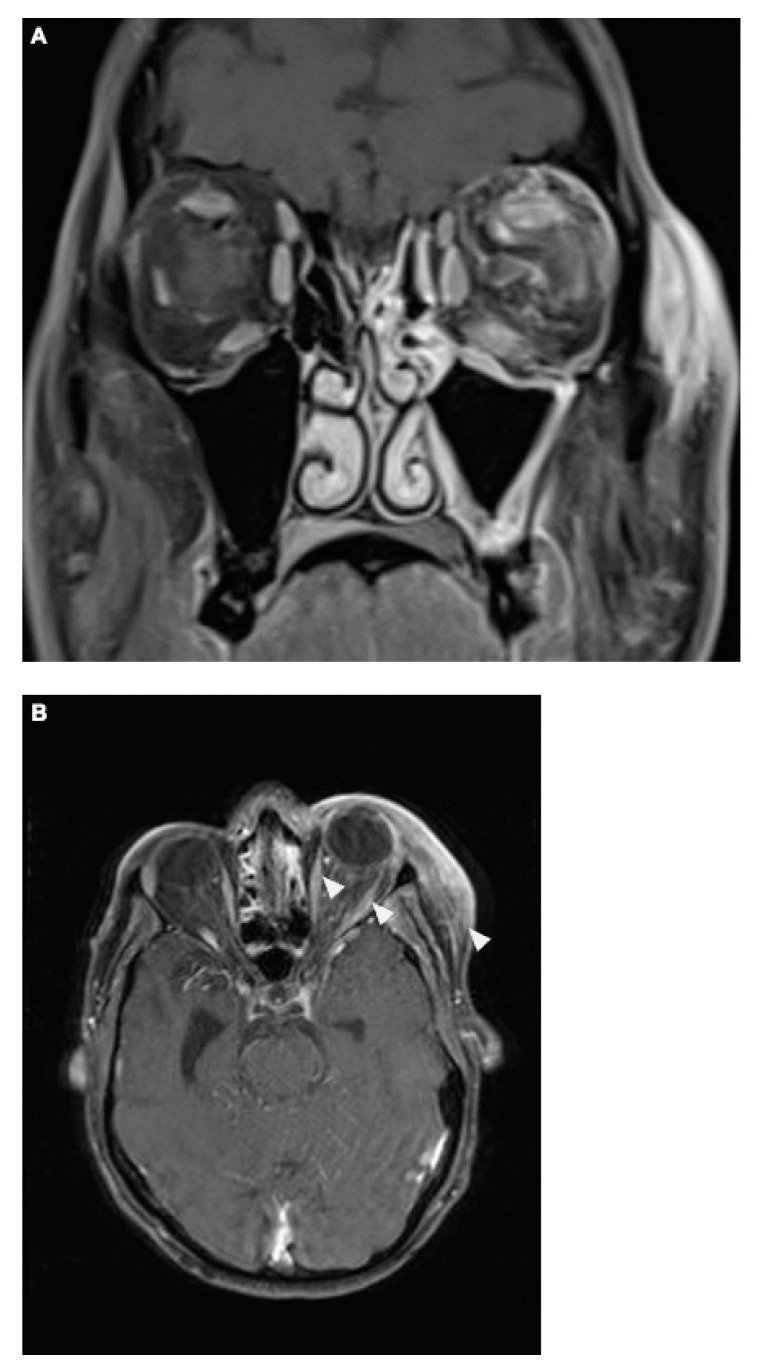
The coronal T1-weighted post-Gadolinium magnetic resonance imaging (MRI) sequence revealed no definitive evidence of cavernous sinus thrombosis. However, venous dilation in the left superior ophthalmic vein was noted, along with significant phlegmonous enhancement at the apex of the left orbit, involving the left superior rectus muscle and potentially the optic nerve. Adjacent sinusitis was observed in the left frontal, ethmoid, sphenoid, and maxillary sinuses, accompanied by mild swelling of the left eyelid and inflammation of the adjacent subcutaneous tissue (**A**). The axial T1 post-Gadolinium MRI sequence (**B**) demonstrated contrast-enhanced protrusion of the left eye muscles, particularly affecting the superior rectus muscle. This indicated ongoing inflammation, though there was a slight reduction in the extent of optic nerve involvement compared to earlier scans, suggesting a partial therapeutic response. Importantly, no signs of cavernous sinus thrombosis were observed (as described above), and the venous structures remained patent.

**Figure 5:**
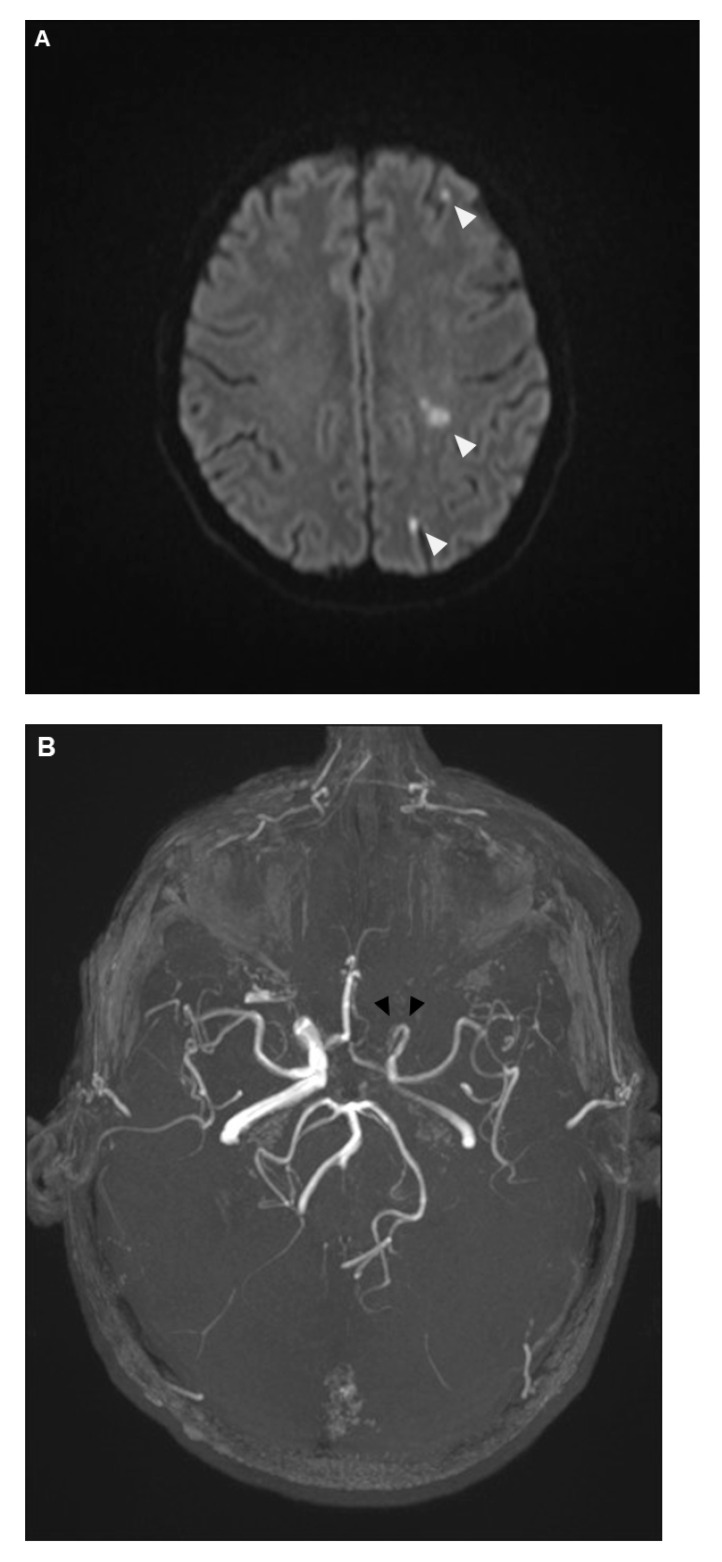

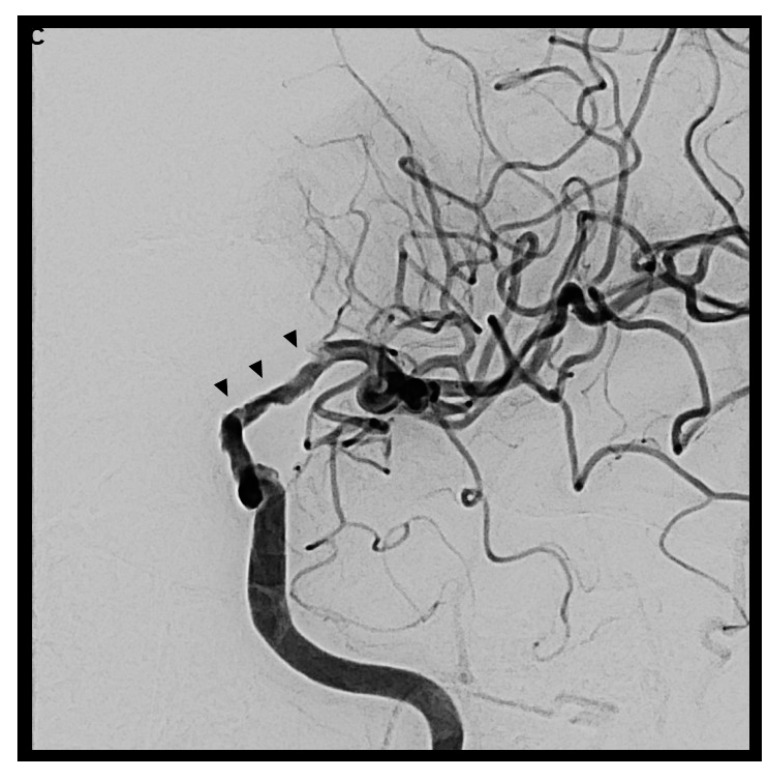
Diffusion-weighted imaging in cerebral MRI showed now new areas of ischemia within the middle cerebral artery territory (**A**). Further evaluation with cerebral MR Angiography (**B**) revealed new, compared to earlier imaging a few days before, irregularities in the vessel walls of the left intracranial internal carotid artery, with moderate-to-severe luminal narrowing. To clarify these findings, Digital Subtraction Angiography (DSA) was performed (**C**). The DSA indicated that while the extracranial segment of the left internal carotid artery appeared normal, significant irregularities were present beginning at the petrous segment. These irregularities (black arrows) caused moderate luminal narrowing with partial filling of the middle cerebral artery from crossflow via the A1 segment. During the venous phase, adequate contrast filling of the cavernous sinus was observed, effectively ruling out a cavernous sinus thrombosis. Despite local administration of 3 mg Nimodipine over 30 min, there was no alteration in the observed vascular irregularities, supporting the conclusion that these changes were inflammatory rather than spasmodic.

**Figure 6:**
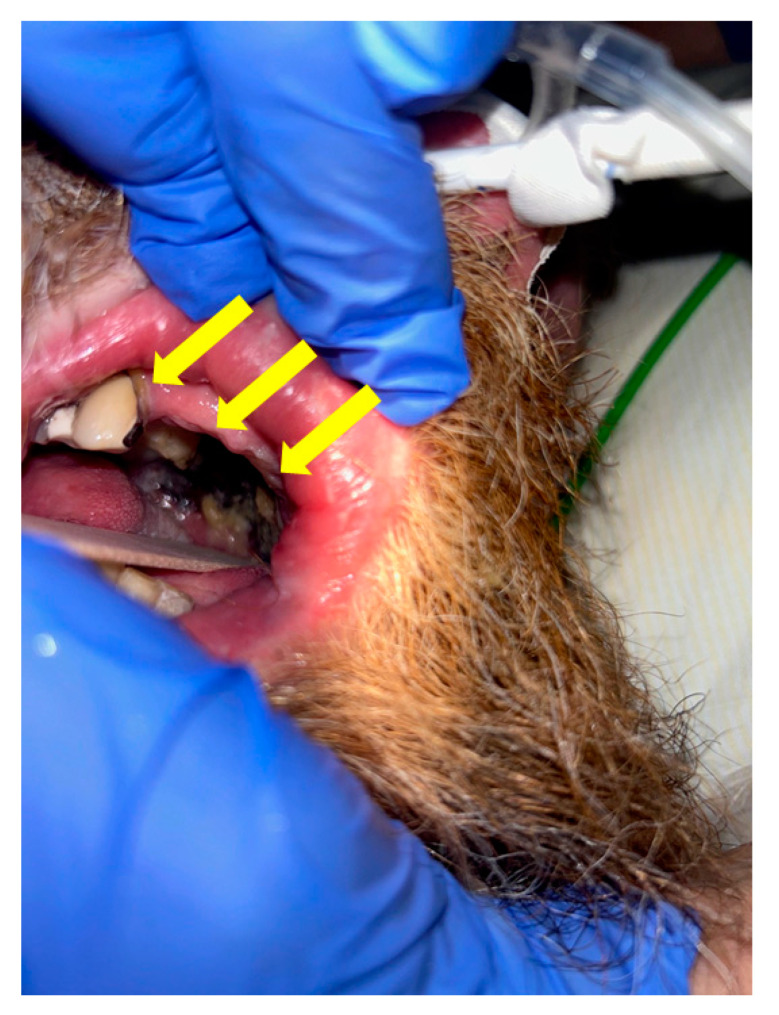
The image displays non-removable blackish-gray discolorations across the palate (yellow arrows), indicative of necrotic lesions that developed on day 9 after initial presentation and day 13 after symptom onset. These findings were suggestive of tissue invasion and necrosis consistent with an invasive fungal infection, likely mucormycosis. Due to these developments, Amphotericin B treatment was initiated.

**Figure 7:**
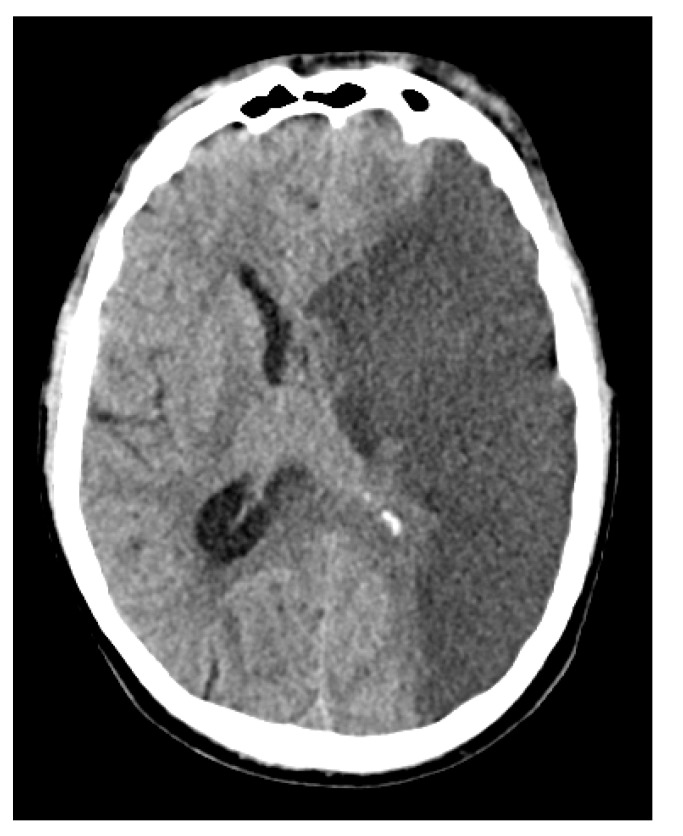
Non-contrast CT scan of the head demonstrating a hypodense area in the left middle cerebral artery territory, indicative of malignant media infarction. The left lateral ventricle is partially compressed, and there is a midline shift of 1–2 cm to the right, suggesting mass effect due to the infarction. No hemorrhagic transformation is seen.
